# Delayed emergence from propofol anesthesia in a patient with Lesch-Nyhan syndrome: A case report

**DOI:** 10.1097/MD.0000000000021847

**Published:** 2020-08-21

**Authors:** Jungwon Lee, Sung Mee Jung, Sungmin Jeon

**Affiliations:** Department of Anesthesiology and Pain Medicine, Yeungnam University School of Medicine, Daegu, Republic of Korea.

**Keywords:** delayed emergence from anesthesia, hyperuricemia, intravenous anesthesia, Lesch-Nyhan syndrome, propofol

## Abstract

**Rationale:**

Lesch-Nyhan syndrome (LNS) is an X-linked recessive disorder presenting with uric acid overproduction, neurocognitive disability, and behavioral disturbances. Inhalational anesthesia has been frequently used in LNS patients undergoing surgery. Characteristic compulsive self-injurious behavior and high risk of emesis may hinder inhalational induction. Propofol may be beneficial for these patients because of its easy and rapid titration for anesthetic depth during induction, early recovery from anesthesia, and antiemetic effect as well as uricosuric effect.

**Patient concerns:**

A 16-year-old male adolescent was scheduled for percutaneous nephrolithotomy. He exhibited poorly controlled muscle, self-injurious behaviors and intellectual disability.

**Diagnosis:**

The patient presented with neurodevelopmental delay in the first year of life, and was diagnosed with LNS, with a substitution of phenylalanine to leucine in hypoxanthine-guanine phosphoribosyltransferase (*HPRT)* 1 gene on the X-chromosome at 3 years of age.

**Interventions:**

Total intravenous anesthesia was used for induction and maintenance of anesthesia with propofol and remifentanil using target-controlled infusion.

**Outcomes:**

Time to recovery of consciousness was prolonged after uneventful surgery. Serum uric acid levels gradually increased during postoperative period.

**Lessons:**

Propofol anesthesia using target-controlled infusion does not provide significant clinical advantages in rapid emergence from anesthesia and management of hyperuricemia in LNS patients undergoing urological surgery.

## 1 Introduction

Lesch-Nyhan syndrome (LNS) is a rare X-linked recessive disorder caused by mutations in the hypoxanthine-guanine phosphoribosyltransferase (*HPRT*) 1 gene, which is involved in the purine salvage pathway.^[1,2]^ It is diagnosed when HPRT enzyme level is <1.5% of normal in males.^[3]^ LNS can fully develop with uric acid overproduction, neurocognitive disability, and characteristic compulsive self-injurious behavior. Uric acid overproduction results in hyperuricemia and hyperuricosuria, which lead to uric acid calculi formation, renal insufficiency, and gouty arthritis. Neurological dysfunction includes developmental delay, cognitive impairment, hypotonia, and extrapyramidal and pyramidal symptoms.^[4]^

Inhalational anesthesia has been frequently used in LNS patients who require surgical procedures.^[5,6]^ Propofol is generally used for sedation in spontaneously breathing patients because of its easy titration for anesthetic depth, rapid recovery from anesthesia, and antiemetic effect.^[7,8]^ Although propofol has been demonstrated to increase urinary uric acid secretion in surgical patients,^[9]^ the preferred anesthetic regimen in LNS patients with hyperuricemia remains unclear. Here, we present a case involving propofol and remifentanil total intravenous anesthesia (TIVA) using target-controlled infusion (TCI) in a patient with LNS undergoing urological surgery. Written informed consent was obtained from the patient and his guardian for publication of this case report and accompanying images.

## 2 Case report

A 16-year-old Asian male adolescent (31.5 kg, 140 cm) with LNS was referred to the urology department for percutaneous nephrolithotomy due to multiple calculi in the right renal pelvis and obstruction of urine flow. He presented with neurodevelopmental delay in the first year of life, and was diagnosed with LNS, with a substitution of phenylalanine to leucine in *HPRT1* on the X-chromosome at 3 years of age. He exhibited poorly controlled muscle movement with axial spasm and hypotonia, self-injurious behaviors and intellectual disability. He was medicated with allopurinol, hydrochlorothiazide, potassium citrate and clonazepam.

Preoperative physical examination revealed moderate intellectual disability, prominent upper front teeth, bruises in the gingiva, and conjunctivitis in the left eye due to poking with his fingers. A spastic quadriparesis was present, making the sitting position impossible without support. Laboratory tests revealed a hemoglobin level of 13 g/dl; serum uric acid, 3.80 mg/dl; serum creatinine, 0.46 mg/dl; serum potassium, 3.2 mEq/L; and urine uric acid, 24.79 mg/dl. This case report was approved institutional review board and the verbal assent and informed consent for publication of his clinical details was obtained from patient and his guardian, respectively.

When the patient arrived to the operating room without premedication, oxygen saturation was 97% with a heart rate of 94 beats/min, noninvasive blood pressure was 127/70 mm Hg, and bispectral index (BIS) score was 94. TIVA was induced using TCI with effect-site concentrations of propofol, 3 μg/ml; and remifentanil, 1 ng/ml. After administration of cisatracurium 5 mg, endotracheal intubation followed by volume-controlled ventilation in 50% oxygen was performed. The effect-site concentrations of propofol and remifentanil were maintained at 2 μg/ml and 0.6 ng/ml, respectively, to maintain BIS scores between 40 and 45 during surgery.

After 55 minutes of uneventful surgery, infusion of propofol and remifentanil was discontinued. Spontaneous respiration immediately resumed. The recovery of train of four from 25% to 75% and to 90% was 4 minutes and 5 minutes, respectively, following reversal of neuromuscular blockade. However, time to recovery of consciousness was 35 minutes, until reaching a BIS score of 81 at a propofol effect-site concentration of 0.7 μg/ml. Propofol and remifentanil were used at 101 μg/kg/min and 0.05 μg/kg/min, respectively, during the 115 minutes of anesthesia.

**Figure 1:**
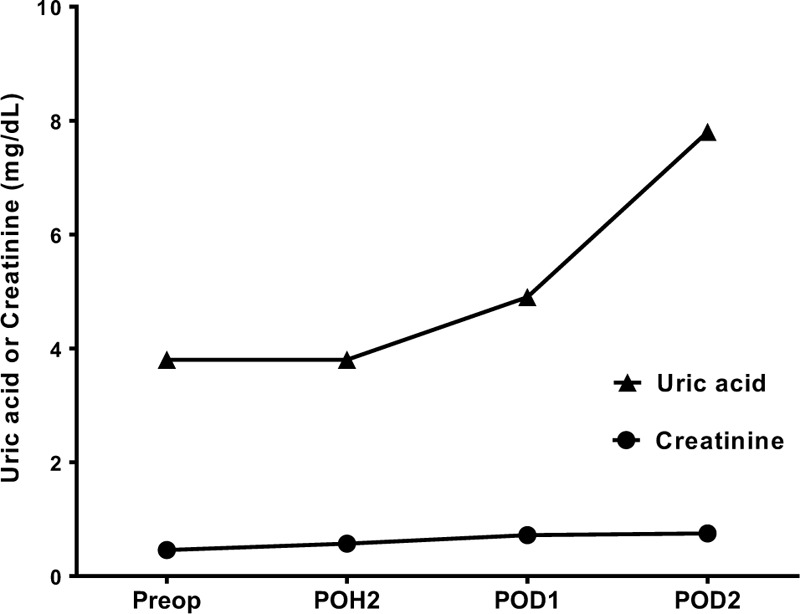
Change in serum uric acid and creatinine levels during the perioperative period. Preop = preoperative day, POH = perioperative hour, POD = postoperative day.

In the postanesthetic care unit, the patient was stable without exhibiting self-injurious behavior. A postoperative pain was successfully managed with intravenous administration of fentanyl 10 μg without nausea or vomiting. Serum uric acid levels gradually increased (normal range, 3.4–7.0 mg/dL) (Fig. 1) despite the administration of allopurinol during postoperative period. He was discharged without complications on postoperative day 3 with carefully increased dosage of allopurinol.

## 3 Discussion

The choice of the general anesthetic regimen in LNS patients depends on results of the preoperative assessment, the type of surgical procedure, primary metabolic defect(s), and the severity of renal impairment. The preoperative assessment should identify anesthetic risk factors that could contribute to poor perioperative outcome(s) in individual patients. Because pulmonary aspiration, laryngospasm, central apnea, and cyanotic breath-holding spells can be lead to sudden life-threatening respiratory events,^[10]^ it is advisable to administer drugs to decrease the volume and increase the pH of the gastric fluid before anesthesia in LNS patients with a history of vomiting. However, metoclopramide (a D2 dopamine receptor antagonist) can worsen extrapyramidal symptoms and dystonia in LNS patients because they also have dopaminergic dysfunction.^[11]^ Before administering general anesthesia, the airway anatomy should also be carefully assessed because repeated destruction and scarification of the oral mucosa, tongue and lips by persistent self-biting increase the likelihood of difficulties in securing the airway such as limitations in mouth opening, airway bleeding, or lack of teeth. Intravascular volume status and electrolyte balance should be also optimized before anesthesia because the concomitant use of diuretics and potassium citrate is common.

Because significant renal impairment in LNS patients may affect the distribution, metabolism, and excretion of commonly used anesthetic agents, short-acting anesthetic drugs not involved in renal metabolism and excretion are preferably recommended. Inhalational anesthetics have been used for the benefits of its low tissue solubility and low rate of biotransformation, particularly in the kidney.^[5,6]^ However, it may be difficult to administer inhalational anesthetics in an individual exhibiting impulsivity and compulsive self-injurious behaviors. In addition, dystonia in severe cases may complicate positioning and airway management. They are also vulnerable to pulmonary aspiration and laryngospasm from gastric contents during mask induction due to a high prevalence of emesis. Data regarding anesthetic management of LNS patients with TIVA with propofol and remifentanil using TCI are limited. The metabolism and excretion of propofol is known to be unaltered by renal failure.^[12]^ Remifentanil is of particular benefit for LNS patients due to its very rapid metabolism by blood and tissue esterase. Therefore, TIVA with propofol and remifentanil may be appropriate in that it enables rapid and smooth induction and emergence, with precise titration of anesthetics when guided by anesthetic depth monitoring.

However, our patient demonstrated a delayed emergence at a lower awakening propofol concentration than would be expected despite meticulous titration of propofol dosage with BIS monitoring.^[13]^ Neither remifentanil nor cisatracurium appeared to affect the pharmacokinetics of propofol in our patient due to the minimal use of remifentanil and immediate recovery of spontaneous respiration after the discontinuation of drugs. We used TCI of propofol according to the Marsh pharmacokinetic model, which scales volumes of the central and peripheral compartments linearly to patient weight. Extremely low lean body mass may affect the predictive performance of pharmacokinetic models, leading to the relatively high propofol dose and delayed emergence in this case. In addition, propofol suppresses arousal-promoting dopaminergic neurons in the brain,^[14]^ which can contribute to delayed emergence from anesthesia in patients with dopamine deficiency diseases such as LNS.^[11]^ Thus, a commercially available TCI model may be inadequate to predict the distribution and clearance of propofol in LNS patients.

In addition to its antiemetic action, uricosuric effect of is assumed to be beneficial for LNS patients undergoing general anesthesia.^[6,9]^ However, no reduction in serum uric acid levels in the postoperative period was observed in our patient. The ineffectiveness of propofol may be explained as follows. HPRT deficiency results in uric acid overproduction, leading to hyperuricemia and subsequent hyperuricosuria, which consequently augments uric acid crystal formation in the urinary system. Therefore, drugs that reduce uric acid production, such as allopurinol, rather than uricosuric drugs are recommended in LNS patients.^[3]^ Thus, propofol does not appear to be of benefit in managing hyperuricemia in LNS patients undergoing surgery.

In conclusion, propofol and remifentanil TIVA using TCI does not offer significant clinical advantages in rapid emergence from anesthesia and management of hyperuricemia in LNS patients undergoing urological surgery. Adjusting pharmacokinetics according to individual patients may facilitate the safe administration of propofol anesthesia by improving the precision of TCI based on appropriate monitoring of anesthetic depth in these patients.

## Author contributions

**Data curation:** Jungwon Lee, Sungmin Jeon.

**Conceptualization:** Sung Mee Jung.

**Supervision:** Sung Mee Jung.

**Writing – original draft:** Jungwon Lee.

**Writing – review & editing:** Jungwon Lee, Sung Mee Jung.
